# Use of Electronic Loggers to Measure Changes in the Rates of Hand Washing with Soap in Low-Income Urban Households in India

**DOI:** 10.1371/journal.pone.0131187

**Published:** 2015-06-23

**Authors:** Richard L. Wright, Ruediger Zillmer, Adam Biran, Peter Hall, Myriam Sidibe

**Affiliations:** 1 Unilever R&D Port Sunlight, Bebington, Wirral, United Kingdom; 2 London School of Hygiene & Tropical Medicine, London, United Kingdom; 3 Intertek Testing Services, Capenhurst, Wirral, United Kingdom; 4 Unilever Kenya, Nairobi, Kenya; Örebro University, SWEDEN

## Abstract

We evaluated the utility of electronic loggers to measure the effects of a simple intervention designed to influence the rates of hand washing with soap within enclosed toilets and bathrooms in low-income urban households in Kerala, India. 58 households were given three items with embedded electronic loggers for a period of 2-5 days. Two logged soaps tracked hand and body washing in the bathroom. The third logged item was a water vessel used for flushing the toilet and for post-defecation anal cleansing; this served as a marker of toilet use. In addition, 28 households in a *Soap by toilet* arm were given an additional logged soap, to be kept by the toilet, and used for hand washing. Compared with the *Soap in bathroom arm*, the loggers in the *Soap by toilet* households recorded 73% greater daily use of soaps designated for hand washing (t(36)=2.92, p<0.01) and 172% greater use within 2 minutes of the use of the water vessel (t(36)=3.51, p = 0.001). We conclude that the loggers were capable of detecting changes in the rates of hand washing with soap and changes in hand washing with soap after use of the toilet. Further adoption of logger technologies would enable more insightful studies of hand washing within urban environments.

## Introduction

There is considerable evidence that hand washing with soap (HWWS) can reduce the incidence of diarrhoeal disease [1–3] and respiratory infections [1, 4]. However, globally, rates of HWWS remain low and there is a pressing need to develop new, effective interventions to increase handwashing practices [5].

A major issue for the field is that measuring handwashing is difficult [6]. In order to evaluate hygiene promotion interventions we need to develop better methods for assessing their behavioural outcomes [6, 7].

The current ‘gold standard’ for measuring handwashing rates is structured observation. However, this is quite intrusive and limited to short periods of time during a day. Further, observation may affect the frequency of the behaviour itself, risking introducing bias into the data [8], and is particularly problematic within urban households, where handwashing and defecation occur within enclosed bathrooms and toilets.

An alternative method for measuring HWWS is to use electronic motion detectors (loggers) embedded in soap bars. Loggers record the time, duration and direction of soap movements thus giving an indication of soap use. Logged soap may be ideal for urban contexts as it is a relatively unobtrusive method that can capture behaviour 24 hours a day and can be used within enclosed bathrooms. However, to date, studies using loggers have been limited to rural environments [8,9] and registered only overall levels of HWWS. They have not been able to determine whether changes in hand washing occur at critical times, such as post toilet use [6].

The primary purpose of the current study was to test of loggers as a means of collecting data on total HWWS and post-toilet HWWS in an urban setting. Additionally, for half the households we introduced a dedicated soap bar to be kept next to the toilet. Our interest was to evaluate whether the loggers were sensitive to changes in HWWS and post-toilet HWWS generated by this intervention.

## Materials and Methods

### Design

The study had two arms. One arm received two logged soaps for the bathroom (*soap in bathroom* arm), the other received logged soaps for the bathroom plus a dedicated logged soap which was to be kept by the toilet (*soap by toilet* arm). All households received logged water vessels to be used for flushing the toilet and anal cleansing. Households were assigned to each arm alternately on recruitment.

#### Study Setting

The study was carried out in low-income households located in Trivandrum, Kerala and a nearby fishing community. Houses were all permanent structures with enclosed indoor bathrooms and toilets. All had piped water, either to the house or a nearby public standpipe. However, water was only supplied for a limited period each day and households stored water in the house for domestic use. Toilets were predominantly pour-flush, connected to sewer systems or septic tanks. All households owned at least one soap bar that was used for personal washing. Soap was rarely stored in toilets when separated from bathrooms, suggesting that HWWS and body washing were not usually practiced in this area.

#### Sampling

Household visits by fieldworkers were used to recruit a convenience sample of 30 households in each of the two communities. All households included at least one child, aged between 6 and 11 years, who was attending a Government-aided or Government school. This criterion was included as these households were of broader interest to Unilever’s Lifebuoy brand, which runs hand washing promotion activities in schools.

#### Loggers

The loggers used in this study have been described in detail elsewhere [9, 10]. They measure 47mm x 17mm x 14mm and are small enough to be embedded within soap bars. Each logger contains a set of sensors including a 3-dimensional accelerometer, acoustic sensor, tilt sensor, real-time clock and a memory chip. These sensors were electronically calibrated during manufacturing and tested in the lab before use.

When used in trials loggers are inductively charged and programmed using a bespoke hub. During the trial loggers remain in low power mode until activated by movement. Once activated they record the date and time, acceleration, and an acoustic signal within a specified frequency band.

Logged soaps were made by warming a 150g soap bar in a microwave oven. The warmed soap was cut in half and recesses the size of the logger cut in each of the two halves. The logger was then placed inside the recesses and the two halves pressed together using a manual soap press.

Logged water vessels were made by attaching a logger to the inside base of a 1 litre white opaque plastic bottle using waterproof glue.

### Procedure

Following consenting, we removed all soaps that were in general family use in the bathrooms of every household. Soaps kept elsewhere in the house and soaps used exclusively for medical purposes, by elderly family members or for babies, were left in place.

In every house we placed two bars of unbranded soap in the bathroom, one red and one white. Each soap contained an embedded logger and weighed approximately 145g. Householders were instructed in their local language of malayalam to use the white soap exclusively for bathing and the red soap exclusively for hand washing.

In the *soap by toilet* arm we placed an additional logged, red soap bar close to the toilet. Householders were instructed to use this soap exclusively for hand washing.

We asked householders to identify the water vessel they would usually use for toilet flushing and anal cleansing following defecation. These were removed and replaced with the logged water vessels.

Logged items were left in households for a period of between 2 and 5 days. During this time houses were visited once for a short observation period, the results of which are reported in Zillmer et al. [10].

On completion of the study fieldworkers retrieved the logged items and returned the original soap bars and water vessels. The remaining soap was weighed and loggers retrieved.

#### Outcome measurement

Data from all households were collected for the purposes of the current study and for validating the loggers’ accuracy at detecting genuine soap use and discriminating HWWS from bathing. Outcomes related to this validation were reported in Zillmer et al. [10]).

Here we estimated the number of ‘HWWS events’ using the raw logger data from the red soaps. First, bespoke algorithms used statistical features based on event duration, sound-level quantile, and acceleration to discriminate between data generated by HWWS and data generated by general movements, unrelated to HWWS. These statistical features were then passed on to a cluster analysis and reference data from laboratory experiments used to identify the cluster that contained HWWS events (see [10] for more details). The number of ‘HWWS events’ was then calculated by combining HWWS occasions separated by less than 60 seconds. This avoids over-counting events due to ‘quiet’ periods in washing routines when soap is not used.

The logged water vessels were used as the indicator of toilet use. The algorithm applied to the raw data identified movement sequences that incorporated inversion of the vessels. This was assumed to indicate the use of the vessel for anal cleansing or flushing of the toilet.

Two main outcome measures were calculated in this study, ‘total HWWS events per household per study day’ (from 1 red bar in the *soap in bathroom arm*, and 2 red bars in the *soap by toilet arm*) and the ‘probability of HWWS events post-toilet’. The latter was defined as the per-household proportion of toilet-uses that were associated with a HWWS event. We considered toilet uses and HWWS events to be associated when they were within 2 minutes of each other.

In addition, we calculated the number of ‘white soap events per household per day’ and ‘probability of white soap use post-toilet’ to establish whether the presence of soap by the toilet affected the use of this soap. The use of the white soaps were most likely to reflect body washing but could include hand washing where instructions were not followed. As with the red soap loggers, data from the white soap loggers were analysed using algorithms and events within 60 seconds of each other combined into single events.

#### Ethics and consenting

All householders were shown a logger. It was explained to householders that these had been inserted into the study soaps and water vessels to record the times when these items were moved. Informed consent forms were written in malayalam and were signed by the head of the household. Consent forms were read to signees when they could not read.

A payment of 150 INR was made to heads of households on completion of the study. The study protocol was approved by the ethics committee of The London School of Hygiene & Tropical Medicine. Local clearances were obtained by the Socio Economic Unit Foundation, Trivandrum, who also assisted with the field work.

#### Data management and analysis

After signal analyses, event data were transferred to JMP for analysis where T-tests were used to compare the two study arms. In order to perform t-tests on the ‘probability of HWWS using after toilet use’ and ‘probability of using white soap after toilet use’, we transformed the raw probabilities using an ‘Empirical Logit’ transform, where $Logit(Ρe)=Log(\frac{Ρe}{1-Ρe})$ [11]. The ‘Empirical logit’ is similar to a Logit transform but uses modified probabilities, pe to remove zero values. pe = (n+0.5)/(N+1).

## Results

Loss to follow-up occurred for a variety of reasons not associated with logger functionality (drop-outs, administrative errors). The final sample included data from 38 households (18 in the *soap by toilet* arm and 20 in the *soap in bathroom arm*) collected over a mean period of 3.95 days (range 2–5 days). The characteristics of these households are shown in Table 1.

**Table 1:** Household characteristics.

		*Soap in Bathroom* (n = 20)	*Soap by Toilet* (n = 18)
**Mean number of occupants by age**	5 and under	0.3	0.3
	6–11 years	1.35	1.5
	12–20 years	0.35	0.5
	Adults (21+)	2.7	2.4
	All ages	4.7 (M = 2.0;F = 2.7)	4.8 (M = 2.1;F = 2.7)
**Toilet flush**	Pour flush	20	17
	Cistern flush	0	1
**Bathroom layout**	Separated	4	3
	Adjoining	14	10
	Single	2	5

Table 2 shows the daily means of HWWS and toilet use as well as the probabilities that toilet use is accompanied with soap use. Statistical tests showed a small but non-significant difference between the arms in term of mean number of toilet uses per day (t(36) = 1.31, p = 0.2). However, during the study period, households in the *soap by toilet arm* showed a greater total number of HWWS events per day (t(36) = 2.92, p < 0.01) and a greater probability of HWWS associated with using the toilet (t(36) = 3.51, p = 0.001). There were no statistical differences between the two arms with respect to the daily rate of white soap use (t(36) = 0.13, p >0.2) or the probability that white soap would be used after the toilet (t(36) = 1.42, p = 0.16).

**Table 2:** Soap and toilet use.

*Outcome*	*Soap in Bathroom (n = 20)*	*Soap by Toilet (n = 18)*	*Ratio*
Mean number of toilet uses per day	4.20	3.37	0.80
Mean HWWS per household per day	6.02	10.39	1.73
Mean probability toilet use is accompanied by HWWS	0.065	0.177	2.72
Mean use of white soap per household per day.	6.72	6.92	1.03
Mean probability toilet use is accompanied by white soap use	0.075	0.108	1.43

## Discussion

In this study, we demonstrated that electronic loggers can be used in an urban setting to measure overall HWWS and post-defecation HWWS. This extends their use beyond measuring only overall HWWS in rural settings, reported elsewhere [8, 9].

Further, we found that our measure was sensitive to a handwashing intervention. Households that received the additional soap beside the toilet showed a 73% increase in overall daily HWWS and a 172% increase in the probability of post-toilet HWWS. There are several possible explanations for this. The soap may have provided a visible reminder to HWWS, the convenience of the soap may have removed a barrier to HWWS, or the increase in the overall availability of soap may have led to more liberal use. These explanations are not mutually exclusive and we are not able to tease them apart.

The study also supports the use of the location of the soap as a proxy for hand washing. Previously, Luby et al. [12] found that soap availability next to the toilet was associated with higher levels of observed post-defecation HWWS, while Halder et al. [13] reported similar relationships with hand cleanliness of adult caregivers and children. However, Luby et al. and Halder et al. both use correlational designs, hence factors that covary with soap availability, such as wealth, could also have caused observed differences in HWWS. Because the present study took an experimental rather than a correlational approach, it provides stronger evidence of a causal link between the placing of soap by the toilet and HWWS.

The combined probability that the usage of red or white soap was associated with jug use was relatively low for both arms; for the *Soap by toilet* arm this was 0.285 compared to 0.14 for the *Soap in bathroom* arm. This should not be taken as an accurate reflection of post-toilet soap use for these households. The use of the water vessels will be associated with the occurrence of defecation and urination, however we would expect that they could also be used during general cleaning. Therefore, these probabilities may under-estimate post-toilet hand and body washing and should be treated as relative scores.

### Limitations

In this study we used acceleration data in combination with acoustic data to classify whether soap had been used for hand washing. These classifications will be subject to errors of commission (identifying HWWS when none occurred) and omission (missing HWWS that actually occurred). However, the work reported in Zillmer et al. [10] indicates that the level of error is not sufficient to explain the changes in we observed.

A second limitation, common with many behavioural studies, was that it was not possible to ‘blind’ either the participants or those responsible for data collection. We expect that the lack of blinding of data collectors did not affect the outcome of the study. The loggers were blind to the status of the household (as were the statisticians) and there were no opportunities for data collectors to affect the integrity of the data due to subjective biases.

As with any behavioural study, it is possible that behaviour in both arms was not representative of behaviour under normal conditions: This may affect both the rates of water vessel use and soap interactions. We attempted to reduce the ‘demand characteristics’ of the situation through ensuring participants were not aware of the experimental hypotheses. However, it is possible that the placement of the additional logged red soap by the toilet put these participants under further pressure to use it to wash their hands following defecation. A final limitation is that we studied behaviour over a relatively short period. After this time there is the possibility that soap bars wear out, exposing loggers. While this time period compares very favourably to structured observation, we were unable to study whether there were longer-term effects of the availability of soap on HWWS.

### Implications

The study suggests that electronic loggers may be an important tool in enabling future research on HWWS where enclosed toilets and bathrooms are used. These technologies have been around for some years and continue to become smaller, cheaper, and more reliable. However, they are not widely used in large behavioural trials outside Unilever and we contend that greater focus needs to be placed on their further adoption.

The effect of the availability of soap on HWWS and post-defecation HWWS warrants further research to determine whether it can be extrapolated to other populations and beyond the four day window of time covered by this study.

## Supporting Information

S1 File Household composition data. (XLSX)

S2 File Logger data. (XLSX)
